# Small Bowel Injury Due to Electrocautery Excision of Umbilical Granuloma in a Four-Month-Old Child

**DOI:** 10.7759/cureus.24940

**Published:** 2022-05-12

**Authors:** Harshal Tayade, Kiran Khedkar, Yashwant Lamture, Jay Dharmashi

**Affiliations:** 1 General Surgery, Jawaharlal Nehru Medical College, Datta Meghe Institute of Medical Sciences, Wardha, IND

**Keywords:** post-electrocautery bowel injury, infant umbilical granuloma burn injury, small bowel injury from electrocautery, electrocautery bowel burns, thermal bowel injury

## Abstract

Many surgeons are familiar with small bowel perforation-a breach in the continuity of the bowel wall resulting in spillage of contents into the peritoneal cavity. Usually, patients present with severe abdominal pain, and radiological investigations suggest pneumoperitoneum. However, intestinal perforation secondary to electrocautery used for umbilical granuloma excision is rare. We report a case of a 4-month-old boy who presented with primary concerns of constipation, severe abdominal pain, and multiple episodes of vomiting three days following an electrocautery excision of umbilical granuloma. An exploratory laparotomy revealed a perforation of the terminal ileum. Primary repair of the ileal perforation was done, which saved the infant’s life. As this case illustrates, even a minor surgical procedure can lead to a major intraperitoneal injury, and appropriate evaluation based on clinical signs and symptoms is imperative. This case is also a reminder to handle an electrosurgical instrument with proper skill, training, and technical assistance.

## Introduction

An umbilical granuloma is an overgrowth of granulation tissue formed during the healing of the umbilical stump after the umbilical cord is separated. It is the most common umbilical abnormality found in infants, occurring in one in 500 newborns [1]. The overgrowth is associated with fibroblasts, pro-inflammatory and inflammatory cells, and endothelial cells, all embedded in edematous stromal tissue [2]. The presentation is a small nodular growth located at the base of the umbilicus. Silver nitrate application is the treatment of choice and has good results [3]. However, some granulomas may persist in childhood and have an intractable nature. Local excision is considered the treatment of choice in persistent, recurrent, and intractable granulomas. Other procedures such as electrocautery, cryocautery, and double ligation have also been used successfully [4]. Fahmy recommends electrocautery by bipolar under anesthesia as the treatment of choice for all cases, stating that the advantages are precise total excision and secure hemostasis with a sample available for histopathology [1]. However, if not used with due caution and skill by a trained surgeon, electrocautery can lead to complications like inadvertent bowel injury accompanied by high morbidity.

## Case presentation

**Figure 1:**
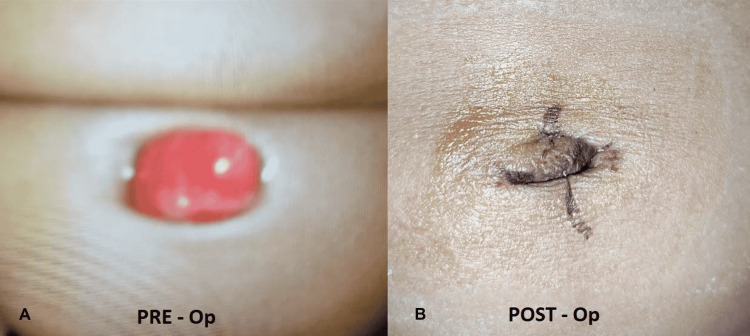
(A) Preoperative image (blurred, supplied by the patient’s family) and (B) postoperative image of umbilical granuloma excised by electrothermal cautery

A 4-month-old male child, with good general health, had been operated on at a single-practitioner, private healthcare unit for umbilical granuloma, wherein excision was done three days prior to the presentation via electrocautery, most likely unipolar electrocautery. Figure 1 presents preoperative and postoperative images.

The child was referred to the emergency unit of a tertiary care center with concerns of fever lasting one day, acute abdominal pain, and multiple episodes of vomiting. There was no history of any illness or trauma prior to the procedure. The child was febrile, with a temperature of 101° F, and irritable, crying excessively, with a pulse of 160 beats per minute and tachypnoea. His abdomen was distended and tender, and he displayed guarding. We noted a few cautery burn marks near the umbilicus. There was no palpable hernia. An erect X-ray of the abdomen was suggestive of pneumoperitoneum. Given his history of prior surgical intervention and clinical and imaging signs, we decided to proceed with an emergency laparotomy by a team consisting of the pediatric surgeon, general surgeon, pediatrician, and pediatric anesthesiologist, with backup support from the pediatric intensive care unit (ICU).

**Figure 2:**
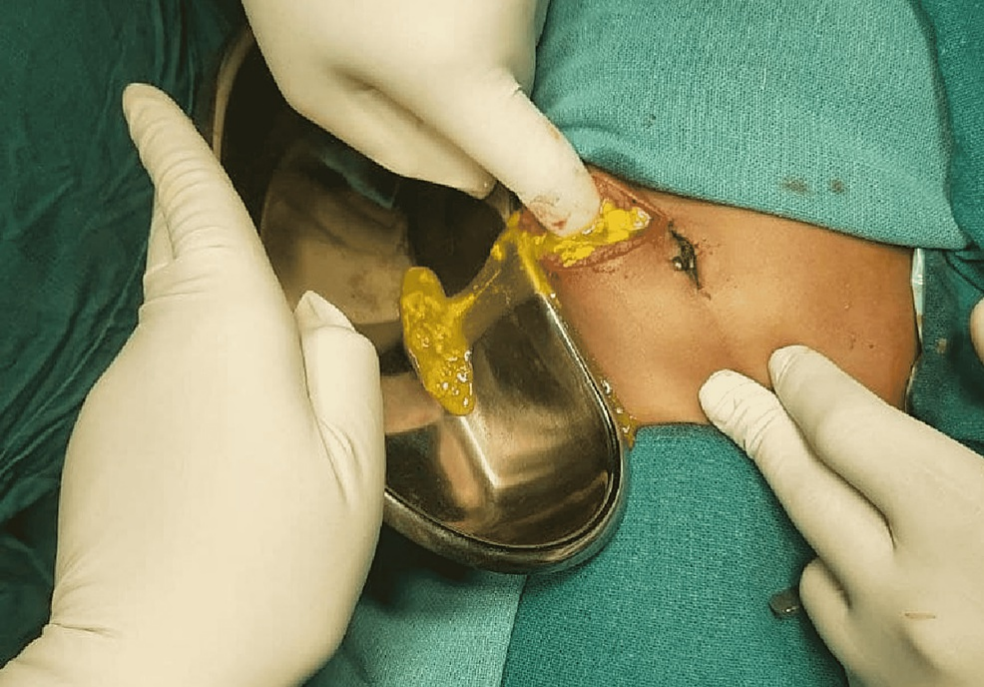
Intraoperative image showing gross contamination of peritoneal cavity with fecal matter

**Figure 3:**
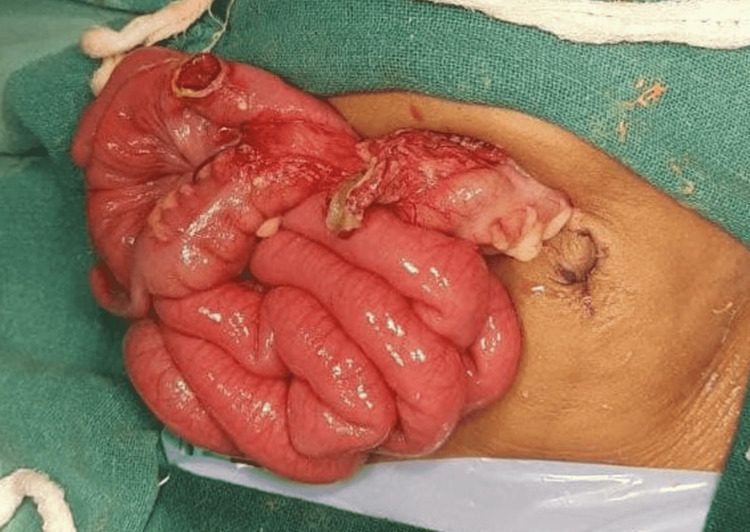
Intraoperative image showing ileal perforation due to thermal bowel injury

An exploratory laparotomy revealed fecal contamination of the peritoneal cavity (as seen in Figure 2), and we noted severe peritonitis and an ileal perforation (as seen in Figure 3) measuring 1 cm × 1 cm, approximately 30 cm from the ileocecal junction.

There was no evidence of vitello-intestinal duct remnants. We performed a double layered, primary repair of the ileal perforation. The procedure was uneventful; however, during the postoperative period, the patient had a fever for 48 hours which was managed with broad-spectrum antibiotics and antipyretics.

The child developed a burst abdomen on postoperative day eight, for which a secondary closure of the abdomen was done with tension sutures. Mesh or skin graft use was not required. The child needed care in the ICU with a multidisciplinary team, appropriate antibiotics, dietary supplements, and supportive care. He recovered and was discharged home.

## Discussion

Bowel injury after excision or electrocautery of umbilical granuloma in a child is rarely seen. A search of the PubMed Index or Google Scholar does not report a single such article [5,6]. These injuries are mostly reported in laparoscopic surgeries due to direct, inadvertent contact capacitance coupling. In the present case, umbilical granuloma was excised using monopolar electrocautery, and the site of perforation was very close to the umbilicus, indicating a deep cautery thermal injury.

The electric cautery method is now rarely used, and the application of silver nitrate is the usual treatment [7]. In cases of recurrence or persistence, excision by cold knife is performed. However, it can be the method of choice in cases of a large granuloma inaccessible to ligation due to technical difficulties. In an infant with a granuloma, the excision site is not under direct vision, and proper control of the cautery point may not be possible; hence extra care must be taken to handle the cauterizing instrument. In handling electrosurgical instruments, care must be taken not to damage the adjacent tissues, and the operating field should be in direct vision. Otherwise, the extent of coagulation obtained would be presumptive. The choice of electrocautery instrument is also crucial. A bipolar device uses a set of forceps; an electrical current passes from one side of the forceps through the target tissue to the other side of the forceps and then back to the generator [8]. Unipolar cautery, however, needs a proper earthing apparatus to ensure the current is grounded [9]. The spillage of current through the body is possible if the return electrode pad is not attached correctly to the patient’s body, especially in an infant or a child.

Using a high- or low-heat cautery point may also be one of the determinants of cautery burns. When the high-heat cautery comes into contact with tissue, charring acts as a protective layer and prevents further destruction of the underlying tissue to a certain extent [10]. With low-heat use, the ultimate destruction of tissue may be far greater, and, as the damage becomes evident after a quiescent postoperative period, the patient may suffer detrimental effects of peritonitis before presenting to the healthcare provider. Thermal injury to the bowel has a hidden depth, causing slow transmural tissue necrosis, and it might also impair local healing and eventually lead to perforation [11]. Thus, the patient may present later than the usual period for wound healing and remodeling as expected. Given the disastrous consequence, performing a good surgical repair of even a minor thermal injury to the bowel is imperative.

## Conclusions

Thermal injury to the bowel in a child undergoing electrocautery excision for umbilical granuloma is rare. This case described a child who underwent a minor procedure and presented with fever and abdominal pain three days postoperatively. As clinical and imaging findings were indicative of perforation, the decision for surgical intervention was evident and life-saving. The classical sign of gas under the diaphragm is diagnostic even in a child. As this case highlights, even a minor surgical procedure can lead to a major intraperitoneal injury, and appropriate evaluation based on clinical signs and symptoms is imperative. Also, handling an electrosurgical instrument requires the proper skill, training, and technical assistance.

## References

[1] Fahmy M (2018). Umbilical Granuloma (UG). Umbilicus and Umbilical Cord.

[2] Ancer-Arellano J, Argenziano G, Villarreal-Martinez A, Cardenas-de la Garza JA, Villarreal-Villarreal CD, Ocampo-Candiani J (2019). Dermoscopic findings of umbilical granuloma. Pediatr Dermatol.

[3] Nagar H (2001). Umbilical granuloma: a new approach to an old problem. Pediatr Surg Int.

[4] Karaguzel G, Aldemir H (2016). Umbilical granuloma: modern understanding of etiopathogenesis, diagnosis, and management. J Pediatr Neonatal Care.

[5] (2022). Search results on PubMed for bowel injury after excision or electrocautery of umbilical granuloma in a child. https://pubmed.ncbi.nlm.nih.gov/?term=Bowel+injury+after+excision+or+electrocautery+of+umbilical+granuloma+in+a+child+.

[6] (2022). Search results on Google Scholar for bowel injury after excision or electrocautery of umbilical granuloma in a child. https://scholar.google.com/scholar?hl=en&as_sdt=0%2C15&q=Bowel+injury+after+excision+or+electrocautery+of+umbilical+granuloma+in+a+child+&btnG=.

[7] Ogawa C, Sato Y, Suzuki C (2018). Treatment with silver nitrate versus topical steroid treatment for umbilical granuloma: a non-inferiority randomized control trial. PLoS One.

[8] Bulsara KR, Sukhla S, Nimjee SM (2006). History of bipolar coagulation. Neurosurg Rev.

[9] Beriat GK, Akmansu SH, Ezerarslan H (2012). The comparison of thermal tissue injuries caused by ultrasonic scalpel and electrocautery use in rabbit tongue tissue. Bosn J Basic Med Sci.

[10] Vedovato JW, Pólvora VP, Leonardi DF (2004). Burns as a complication of the use of diathermy. J Burn Care Rehabil.

[11] Bhullar JS, Gayagoy J, Chaudhary S, Kolachalam RB (2013). Delayed presentation of a bowel Bovie injury after laparoscopic ventral hernia repair. JSLS.

